# Effects of charge fluctuation and charge regulation on the phase transitions in stoichiometric VO_2_

**DOI:** 10.1038/s41598-020-73447-9

**Published:** 2020-10-13

**Authors:** Siddharth Joshi, Nicholas Smieszek, Vidhya Chakrapani

**Affiliations:** 1 Howard P. Isermann Department of Chemical and Biological Engineering, Rensselaer Polytechnic Institute, Troy, NY 12180 USA; 2 Department of Physics, Applied Physics, and Astronomy, Rensselaer Polytechnic Institute, Troy, NY 12180 USA

**Keywords:** Phase transitions and critical phenomena, Electronic devices

## Abstract

Detailed electrical and photoemission studies were carried out to probe the chemical nature of the insulating ground state of VO_2_, whose properties have been an issue for accurate prediction by common theoretical probes. The effects of a systematic modulation of oxygen over-stoichiometry of VO_2_ from 1.86 to 2.44 on the band structure and insulator–metal transitions are presented for the first time. Results offer a different perspective on the temperature- and doping-induced IMT process. They suggest that charge fluctuation in the metallic phase of intrinsic VO_2_ results in the formation of e^−^ and h^+^ pairs that lead to delocalized polaronic V^3+^ and V^5+^ cation states. The metal-to-insulator transition is linked to the cooperative effects of changes in the V–O bond length, localization of V^3+^ electrons at V^5+^ sites, which results in the formation of V^4+^–V^4+^ dimers, and removal of $\pi^{*}$ screening electrons. It is shown that the nature of phase transitions is linked to the lattice V^3+^/V^5+^ concentrations of stoichiometric VO_2_ and that electronic transitions are regulated by the interplay between charge fluctuation, charge redistribution, and structural transition.

## Introduction

Despite the many intensive investigations, the fundamental nature of the non-magnetic insulating ground state of VO_2_ is not well-understood. During a temperature-induced transition that occurs at 340 K, the non-magnetic (NM) monoclinic (M1) ground state undergoes a phase transition to a paramagnetic rutile (R) lattice that is metallic^1^. The prominent theories attribute the insulator-to-metal transition (IMT) process to the structure-driven Peierls-distortion^2^, Mott–Hubbard e^−^ correlation effects^3^, or a combination of both these mechanisms^4–6^. Dopants and time-resolved diffraction studies^7^ lend support to the Peierls-distortion theory, while more recent time-resolved results^8–10^ support the Mott–Hubbard e^−^ correlation. Common theoretical probes like local density approximation (LDA) and generalized gradient approximation (GGA) while correctly predicting the metallic state of the R phase fail to reproduce the insulating state of M1 phase^11^. In contrast, the LDA + U method predicts insulating states with finite band gaps for both M1 and R phases^12,13^. The dynamical mean-field theory (DMFT) and its variants accurately predict the metallic and insulating states of the R and M1 phases^5,14,15^, respectively, however, they are based on adjustable parameters. Similarly, the hybrid Heyd–Scuseria–Ernzerhof (HSE) method correctly predicts electrical phases^16^ but not their magnetic properties^17^. More recently, calculations using modified Becke–Johnson exchange potential (mBJLDA) of DFT have successfully reproduced all the structural, electronic, and magnetic features of the IMT process^18^. However, the failures of common theoretical probes to accurately describe the ground state of VO_2_ raises the question if its chemical nature has been correctly captured in the experimental studies. Interestingly, in a prior study, Boyarsky et al.^19^ noted that the asymmetric ^51^V nuclear magnetic resonance (NMR) spectral line of insulating VO_2_ can be accurately fitted with the superposition of symmetrical lines of V^3+^ and V^5+^, and therefore, suggested that the transition from metallic to insulating ground state involves the transition $2V^{4 + } \to V^{3 + } + V^{5 + }$ with the ground state composed of V^3+^ and V^5+^ ions. However, a subsequent study by Neilsen et al*.*^20^ disputed the analysis of Boyarsky et al. by employing both higher and lower-field NMR measurements, and noting that the insulating phase consists of only V^4+^ cations. In an unrelated study, Chen and Fan^21^ found that charge localization and segregation in the form of V^5+^ can occur at the surface due to subtle fluctuations of deposition conditions which can induce acceptor doping in otherwise n-type VO_2_. One way to elucidate the true chemical nature of stoichiometric VO_2_ is by studying the electronic structure and the related phase transitions in carefully prepared under- and over-stoichiometric phases close to the stoichiometric point without inducing phase change to other vanadium oxide compounds. It is well known that introduction of oxygen vacancy ($\ddot{V}_{O}$) defects in the lattice results in a decrease in the transition temperature, T_IMT_, to room temperature, or below^22^. In this study, we carried out detailed transport and photoemission studies on compositionally-modulated VO_2_ lattices, from VO_1.86_ to VO_2.44_, by systematically introducing oxygen interstitial ($O_{i}$) defects into VO_2_ to understand the effects on oxygen over-stoichiometry on the valence band (VB) structure, electrical resistivity, and structural transitions. Our results shed a new light on the IMT process. They indicate that stoichiometric VO_2_ in the metallic state is multi-valent with co-existing V^3+^ and V^5+^ ions that are a result of charge fluctuation of lattice V^4+^ cations. Localization of charge carriers at the disproportionated cations sites, likely due to strong correlation effects, together with lattice distortions, lead to the nucleation of an insulating phase. Similar multivalence of the stoichiometric lattice have previously been noted in other correlated-systems. A striking example of this occurs in stoichiometric CrO_2,_ a 3d^2^ system with Cr in the + 4 state, which is expected to be a Mott insulator. However, results show that CrO_2_ is a half-metallic ferromagnet due to its mixed valent lattice composed of Cr^3+^ and Cr^4+^ ions^23–25^.

## Results

Measurements were performed with two-dimensional (2D) single crystalline platelets of VO_2_ synthesized from phase transformation of V_2_O_5_ 2D platelets that were grown using hot filament chemical vapor deposition technique (see “Methods”)^26,27^. Scanning electron micrographs (Fig. S1A in the supporting information, SI) showed the platelets to be 1–2 microns in length and 20–50 nm in thickness. X-ray diffraction patterns (Fig. S1B) showed a characteristic 2θ angle at 26.9° corresponding to $\left( {\overline{1} 11} \right)$ monoclinic VO_2_. The as-produced platelets were metallic due to lattice oxygen-deficiency ($VO_{2 - x}$) and were converted to semiconducting, stoichiometric VO_2_ by annealing in air at 220 °C for 72 h or until they showed a characteristic IMT at 67 °C (Fig. S1C). The stoichiometry of this sample, as determined from the ICPMS analysis, was VO_1.97_ (referred henceforth as VO_2_).

Prior studies have shown that non-native dopants, such as W, Nb, Cr, etc. strongly affect the T_IMT_^28,29^. However, their presence introduces additional structural distortion due to lattice strain^30,31^, and gives rise to overlapping chemical and magnetic signatures, thus complicating the study of phase transition behavior. The issues can be avoided if methods exist for the controlled introduction of native defects (V and O) that would then enable the study of resulting band structure changes and its corresponding influence on IMT. While O deficiency can be easily engineered during the growth process because of the propensity of VO_2_ for O vacancies, introducing excess O into the lattice without nucleation of secondary phases such as V_2_O_5_ is challenging, and has not been demonstrated. Initial attempts at controlled increase in the oxygen stoichiometry of VO_2_ through thermal annealing without causing the nucleation of a V_2_O_5_ phase were unsuccessful. Prolonged annealing of VO_2_ samples in air at 220 °C did not increase the O stoichiometry or the room-temperature resistance value, while oxidation at a slightly higher temperature of 250 °C, even for a short duration, resulted in the nucleation of V_2_O_5_ phase within the VO_2_ phase. A faint characteristic Raman peak at 993 cm^−1^ (Fig. S1D) along with the observation of a faint yellow tint of the surface post-oxidation provided confirmation for the incorporation of excess O as a V_2_O_5_ phase instead of uniform bulk doping to give VO_2+y_. Therefore, an electrochemical approach was pursued to modulate the O stoichiometry. The advantage of electrochemical doping is that a controlled number of O dopants can be introduced uniformly across the lattice by simply controlling the total number of injected charge carriers. Prior studies have demonstrated the synthesis of superconducting oxide with a wide range of oxygen over-stoichiometry through electrochemical doping in KOH electrolyte, which was not achievable through oxidative thermal annealing^32–35^. We note that electrochemical doping is distinct from the electrochemical gating process in that the doping is irreversible, (or quasi-reversible) involving oxygen incorporation into the lattice, which is stable even after emersion from the electrolyte and subsequent heat treatment. The key advantages of electrochemical doping over thermal treatment is its ability to charge thick films of nanostructures due to their nanoporous nature, which allows percolation of ions within the film and reduces the time needed to achieve uniform bulk doping.

**Figure 1:**
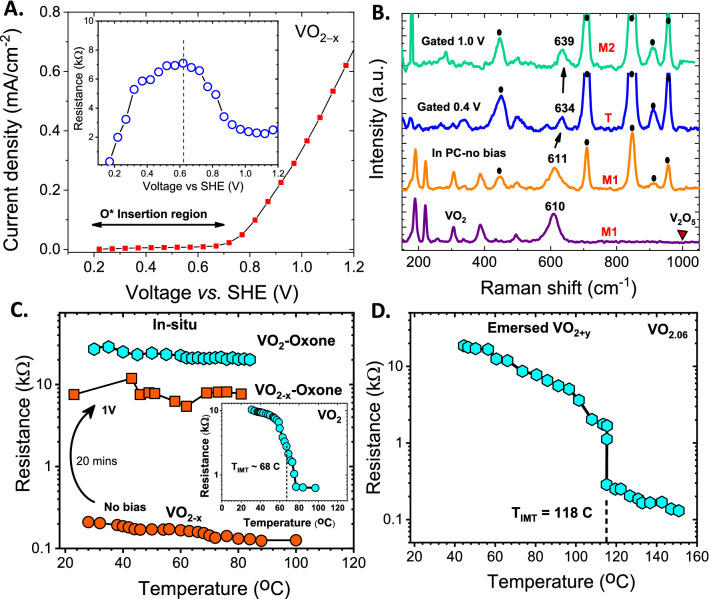
(**A**) Linear voltammogram of $VO_{2 - x}$ electrode during oxidative potential sweep in air-saturated 0.1 M OXONE electrolyte. The potential region of O insertion is indicated. Inset: changes in the resistance of $VO_{2 - x}$ as a function of applied bias showing the transition from metallic phase to oxygen-rich insulating phase; (**B**) Raman spectrum of VO_2_ before and after electrochemical oxidation at various potentials. Increasing positive charging potentials lead to sequential structural phase transitions from M1 to T to M2 monoclinic phases. Peaks denoted by (solid circle) corresponds to the Raman peaks of PC electrolyte; (**C**) changes in the resistance of VO_2_ and $VO_{2 - x}$ as a function of temperature after electrochemical oxidation at 1 V in OXONE electrolyte showing the complete suppression of formation of the metallic phase; (**D**) resistance of dried, emersed VO_2+y_ electrode after electrochemical bias in air.

Given the instability of VO_2_ in KOH electrolyte, oxidation was performed in air-saturated propylene carbonate (PC) electrolyte containing peroxomonosulfate ($HSO_{5}^{ - }$) ions with 1.6% active oxygen (tradename OXONE). OXONE has a higher redox potential (+1.81 V) than KOH (+1.23 V), and, therefore, is a more powerful oxidant than KOH. As a result, a wide concentration range of O stoichiometry from 2 (VO_2_) to 2.44 (V_2_O_5_) was achievable. Figure 1A shows the measured electrochemical response of an oxygen-deficient $VO_{2 - x}$ film during anodic oxidation in OXONE electrolyte. The oxygen doping process was confirmed via simultaneous in-situ measurement of sample resistance (inset of Fig. 1A) during polarization. Data showed that the oxygen insertion occurred at potentials between 0.2 and 1.0 V versus the standard hydrogen electrode (SHE). At potentials greater than 1.0 V, large faradaic currents were observed due to gas evolution that led to a decrease in the amount of inserted oxygen. All doping experiments were, therefore, done within the stable potential window between 0.4 and 1 V (Supplementary Fig. S2).

The structural phase transitions resulting from the doping process were monitored through Raman measurements^8,36^. Samples after polarization were removed from the electrolyte, washed in PC, and dried under vacuum before measurements. Figure 1B shows the Raman spectrum of the VO_2_ film before and after oxidative doping at 0.4 V and 1.0 V. Spectrum of pristine samples showed sharp peaks at 140, 189, 221, 307, 496 and 611 cm^-1^ that are characteristic of pure monoclinic M1 phase^8,36^. A shift in the A_1g_ mode of the V–O bond from 611 to 634 cm^−1^ along with a peak at 589 cm^−1^ confirmed a phase change from M1 phase to a triclinic T phase^36^ for electrodes polarized at 0.4 V. Further polarization at 1 V resulted in the disappearance of the small peak at 589 cm^−1^ and a further shift of the A_1g_ mode to 639 cm^−1^, which is indicative of the formation of the insulating M2 monoclinic phase. The lack of the vanadyl mode at 993 cm^−1^, as well as of the most intense lattice mode at 147 cm^−1^ confirmed uniform doping and the absence of V_2_O_5_ phase in the oxidized samples.

Figure 1C shows the resistance changes of $VO_{2}$ and $VO_{2 - x}$ electrodes as a function of temperature measured after oxidation at + 1 V bias for 20 min without emersion from the electrolyte. Oxidation led to a complete suppression of IMT up to 90 °C in both $VO_{2}$ and $VO_{2 - x}$ . Metallic VO_2_ also showed two-orders of magnitude increase in resistance after 20 min polarization at 1 V bias along with a phase transition from M1 to M2 with no IMT transition up to 90 °C. Measurements in an extended temperature range done on emersed dried films in air showed an IMT at 118 °C (Fig. 1D). This increase in T_IMT_ from 67 to 118 °C with O doping is similar to trends seen with Cr-doping.

### Valence band density of states

**Figure 2:**
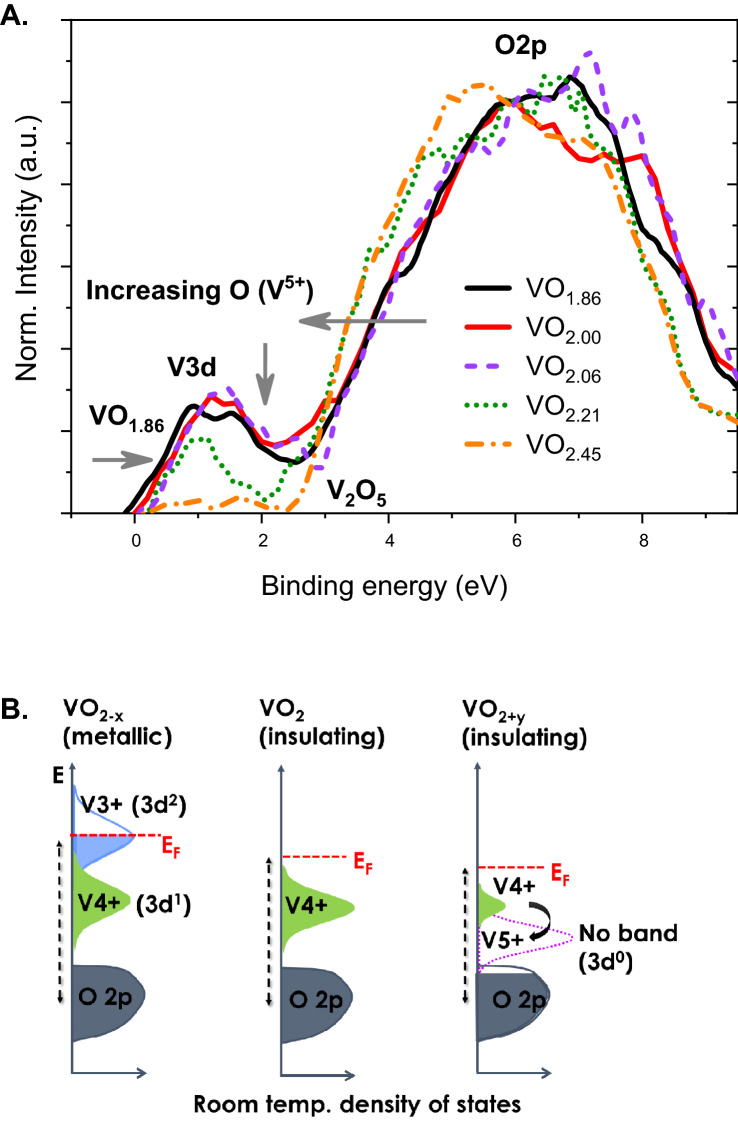
Valence band XPS spectrum of VO_2_ of various oxygen stoichiometry in comparison to spectrum of V_2_O_5_ measured at room temperatures; and (**B**) schematic showing the observed changes in the VB spectrum with varying O stoichiometry.

**Figure 3:**
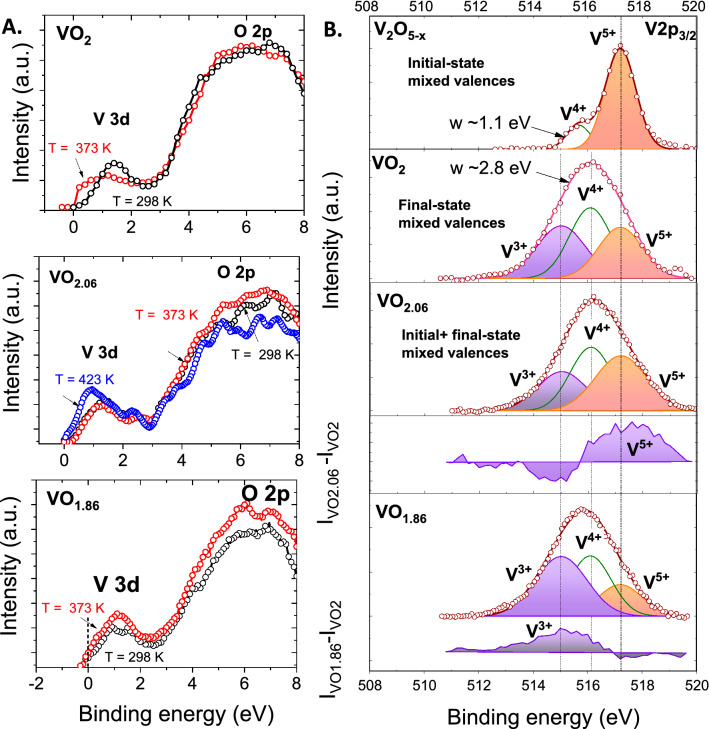
(**A**) Valence band XPS spectrum of VO_2_, $VO_{2 - x}$, and $VO_{2 + y}$ measured at room temperatures (298 K, black curve) and above (373 K, red; and 423 K, blue) IMT transition temperature. (**B**) Comparison of V 2p_3/2_ core-level XPS envelope at room temperature showing the existence of multiple V oxidation states in the initial and final states of various vanadium oxides in comparison with the spectrum of V_2_O_5_. Dots in the figure represent raw XPS data, and thick solid lines represent convolution of individual peaks shown in thin solid lines.

The changes in the electronic structure occurring as a result of O doping was studied through X-ray photoemission measurements. Both stoichiometric and non-stoichiometric samples likely have some V_2_O_5_ on the surface that results from over-oxidation, as noted by Chen and Fan^21^. Therefore, all samples for photoemission studies were prepared by gently sputtering with an Ar^+^ beam for 2 min to remove any over-oxidized surface layer, as monitored via V^5+^ signal in the V 2p core-level peak, and followed by annealing in ultrahigh vacuum at 220 °C for 3 h to desorb physisorbed electrolyte or other contaminants and recover any damage induced by the ion bombardment^37^. Both core-level and valence band (VB) spectrum (Figs. 2, 3) were recorded on these UHV-prepared samples at various temperatures. To observe any systematic trend in the changes in the VB structure resulting from O doping, we compared the room-temperature VB spectra of VO_2_ with increasing O stoichiometry with the spectrum of V_2_O_5_. To enable comparison, the spectra from various samples were normalized to the intensity of the O 2p band centered at a binding energy (BE) of 6 eV, and are shown in Fig. 2. The VB signal comprises of signal from the V 3d band (E_F_ to 2.5 eV) and the O 2p band (2.5–10 eV). The lower energy spectral function seen above E_F_ is generally referred to as the incoherent feature, which arises mainly from the lower Hubbard band (LHB) of V 3$d_{\parallel }$ orbitals^38^. Cluster model calculations of Mossanek and Abbate^39^ indicate that this incoherent feature is due to screening of 3d^1^ electrons by O 2p ligand. The small signal seen at energy at E_F_ in VO_1.86_, represents the delocalized d-band states and represents the coherent part of the spectral function. These signals from V 3d states are nearly absent in the spectrum of V_2_O_5_. The dominant V^5+^ ions of V_2_O_5_ have a 3d^0^ electronic configuration and therefore does not contribute to the photoemission signal in this energy range. The residual signal seen between E_F_ and 2 eV arises from the small amount of V^4+^ ions (Fig. 3B) with 3d^1^ configurations that are formed due to the $\ddot{V}_{O}$ defects in the lattice, as noted in an previous study^26^. With increasing O content of the VO_2_ lattice, three trends become noticeable in the room temperature VB spectra (Fig. 2): (1) The O 2p band progressively shifts to lower BE; (2) The rising edge of the VB curve shifts to higher BE, and (3) The width of the V 3$d_{\parallel }$ band decreases dramatically with increasing O stoichiometry, indicating a lower density of occupied 3d states (DOS). The observed trends are shown in schematically as a band diagram in Fig. 2B. An electrostatics model of the ionic lattice suggests that an increase in the oxygen interstitial ($O_{i}$) defects will lead to an increase in the hole concentration in accordance with the defect reaction in Kröger–Vink notation:$\left[ {\frac{1}{2}O_{{(g)}}  \leftrightarrow O_{i}^{{\prime \prime }}  + 2h^{ \cdot } } \right]$. The trapping of these holes at the nearest-neighbor V^4+^ sites in VO_2_ would create V^5+^ sites with corresponding acceptor-type defect states above the VB of VO_2_. In contrast, donor-type V^3+^ defect states are created from $\ddot{V}_{O}$ defects through the reaction $\left[ {O_{O}^{X} \leftrightarrow \ddot{V}_{O} + 2e^{\prime} + \frac{1}{2}O_{(g)} } \right]$ and e^−^ trapping at the V^4+^ sites. Therefore, it can be concluded that an overall higher DOS and the observed finite number of states at E_F_ (metallic phase) in oxygen-deficient VO_1.86_ is due to the presence of V^3+^-related occupied states (Fig. 2B). This leads to an increase in the E_F_, which manifests as an increased energy separation (5.7 eV) between the E_F_ and O 2p band center in the PES spectrum. On the other hand, increasing holes in the O 2p band with increasing $O_{i}$ defects result in a lower V 3d band width due to the interconversion of part of V^4+^ to V^5+^ with 3d^0^ electronic configuration that does not contribute to PES signal. In an n-type lattice, the increasing hole concentration will result in a lower free electron concentration due to e^−^ trapping which will lead to a decrease in the E_F_ value. In PES spectrum, this results in a decrease in the energy gap between the E_F_ and O 2p band center (5.1 eV). All samples except VO_1.86_ exhibit insulating (semiconducting) behavior. We note that there have been important prior XPS studies^40,41^ on phase transitions in the other various phases of vanadium oxides such as V_2_O_3_, V_3_O_5_, V_4_O_7_, and V_6_O_13_, and has also been reviewed by Surnev et al*.*^42^ These oxides belong to Wadsley (V_2n_O_5n−1_) or Magnéli (V_n_O_2n−1_) phases with increasing O/V ratios. The results of the present study are however distinct from the results of these prior studies in that they represent oxygen composition that are in between the above stoichiometric phases, and thus aid in understanding phase behavior slightly above and below the stoichiometric point of a given crystallographic phase. Additionally, while we observe an increase in the T_IMT_ of VO_2_ with O stoichiometry, this trend is not necessarily followed among the various stoichiometric phases of vanadium oxides. For instance, V_6_O_13_, which has a higher O/V ratio (2.167) than that of VO_2_ (2), has a lower T_IMT_ (145 K) than that of VO_2_ (340 K). The only undoped vanadium oxide that shows a true electronic phase transition at T_IMT_ greater than that of VO_2_ is V_3_O_5_, a lower O/V oxide (1.67), with a T_IMT_ of 425 K^43^.

The variations in T_IMT_ with O stoichiometry seen with temperature-dependent resistance measurements was confirmed using VB spectra obtained at different temperatures (Fig. 3A). In stoichiometric VO_2,_ the V 3d DOS show a clear shift towards the E_F_ at 100 °C, which is higher than T_IMT_ of 67 °C, and thus confirming the formation of a metallic state. During phase transition, the signal of lower-BE V 3d states (coherent states) is seen to increase at the expense of higher-BE V3d states. In over-stoichiometric $VO_{2 + y}$, only a small shift is seen at 100 °C, which is below the T_IMT_ of 118 °C. However, a larger DOS shift to lower BE is seen at 150 °C, which is indicative of the metallic state, and is consistent with the bulk transport measurements. The spectrum of $VO_{2 - x}$ showed finite number of states at E_F_ both at RT and at 100 °C that is consistent with their metallic nature.

### Broadening of V 2p core-level spectra: localized and delocalized ground states

Analysis of the core-level V2p_3/2_ envelope was performed between samples of different oxygen stoichiometry to identify valent states involved in phase transitions, as shown in Fig. 3B. All spectra were corrected for the background signal using Shirley analysis and the peak position and area were optimized by a weighted least-square fitting method using Gaussian line shapes.

No contribution from adsorbed water or other ambient adsorbates, which are seen at BEs of 532.5–533 eV^44^, was observed on UHV-annealed samples. The V 2p_3/2_ spectrum of V_2_O_5_ shows a sharp peak at 517.2 eV that corresponds to the V^5+^ oxidation state. A set of V_2_O_5_ samples were intentionally grown under reduced conditions to generate increasing lattice $\ddot{V}_{O}$ defects in the material. The V 2p_3/2_ spectrum of one such V_2_O_5−x_ sample, also shown in Fig. 3B, shows the presence of an additional peak at a BE of 515.8 eV, corresponding to V^4+^ cations. The full-width-at half-maximum (FWHM) of the V^4+^ defect-related peak ranged from 1.1 to 1.3 eV when present in low and high concentrations, respectively. On the other hand, the V 2p_3/2_ spectrum of stoichiometric VO_2_ shows a broad peak at 516.1 eV, also corresponding to V^4+^, which can be fitted with a single Gaussian peak of FWHM of 2.8 eV, which is within the reported values of 2.0–3.2 eV. We note that the reported V 2p_3/2_ spectrum of both in-situ cleaved or UHV-prepared (similar to current study) single crystalline and polycrystalline VO_2_ are in general broad, as seen from the reported values summarized in Table S1 in SI, and in general much broader than V_2_O_5_ or VO phases. Interestingly, the spectrum of mixed-valent vanadium oxides such as (V_6_O_13_, V_4_O_7_, V_3_O_5_, and V_2_O_3_) that exhibit IMT at varying temperatures are similarly broad. In general, peak broadening occurs due to both intrinsic and extrinsic mechanisms^45^. Apart from the known effects of the instruments limitations or core–hole lifetime changes, important broadening mechanisms of core-level peaks, especially in open-shell systems like VO_2_ can be attributed to (1) multiplet features arising due to the angular momentum coupling between the core–hole created due to photoionization and open-shell valence d electrons, and (2) phonon broadening. However, the multiplet splitting expected to be present in VO_2_ cannot explain the magnitude of observed broadening because multiplet effects on V^4+^ cation in a reduced V_2_O_5_ lattice (V_2_O_5−x_) gives rise to a peak broadening of only 1.1–1.3 eV (FWHM), which is much less than the 2.8 eV broadening seen in VO_2_. Similarly, if the dominant mechanism for broadening involves phonons, one would expect to see similar broadening of the O 1 s peak, which is not observed. Therefore, a different mechanism must be operative. Sawatzky et al.^46,47^ attributed the broadening to “valence fluctuation” due to the stronger core–hole valence–electron interaction (Q) compared to valence electron band width (W). Typical values of Q in vanadium oxides is 0.8–1.2 eV, which is obtained by comparing the typical BE shifts between different oxidation states of vanadium. In comparison, the band gap, d–d separation, and the band width are 0.6 eV, ~ 1.0 eV and 1.5 eV, respectively. Therefore, electron excitation from the filled $d_{\parallel }$ band to conduction band (CB) states in order to screen the influence of photoionized core-holes can result in bound e^−^ (V^3+^) and h^+^ (V^5+^) pairs that would correspond to instantaneous mixed-valent final states^47^. Correspondingly, the single broad Gaussian peak in VO_2_ can be resolved to three Gaussian peaks corresponding to V^3+^ (515.0 eV as in V_2_O_3_) V^4+^ (516.1 eV), and V^5+^ (517.2 eV as in $V_{2} O_{5}$) final states. The ratio of V^3+^ to V^5+^ is ~ 1. It is important to note that such states are not representative of chemical shifts occurring in the sample (initial state)^48,49^, which remains in the V^4+^ state. This is consistent with the average vanadium oxidation state of + 3.93 determined from ICP-MS analysis (Supplementary Table S3).

In non-stoichiometric VO_2,_ a further broadening of the 2p peak is observed. An increase in O stoichiometry gives rise to an increase in the signal at the BE corresponding to V^5+^, as seen from the difference spectrum (ΔI versus BE curve), and is as expected because hole doping should increase the number of V^5+^ cations in the lattice. In contrast, the spectrum of VO_2−x_ shows an increase in the signal at a lower BE corresponding to the signal of V^3+^, which is consistent with the chemical shift expected from the presence of V^3+^ in the lattice due to the formation of $\ddot{V}_{O}$ defects. Here, the 2p_3/2_ peak broadening represents both initial (chemical shift) and final (photoionized) mixed-valent states. This is further confirmed by comparing the average oxidation state estimated through this XPS analysis of the relative area contribution of the individual peak to the overall spectrum to the average vanadium oxidation state calculated from ICP-MS data. For $VO_{2 - x}$, the analysis gives the average oxidation state of + 3.76, which is relatively close the value of + 3.72 from ICP-MS analysis (Supplementary Table S3).

**Figure 4:**
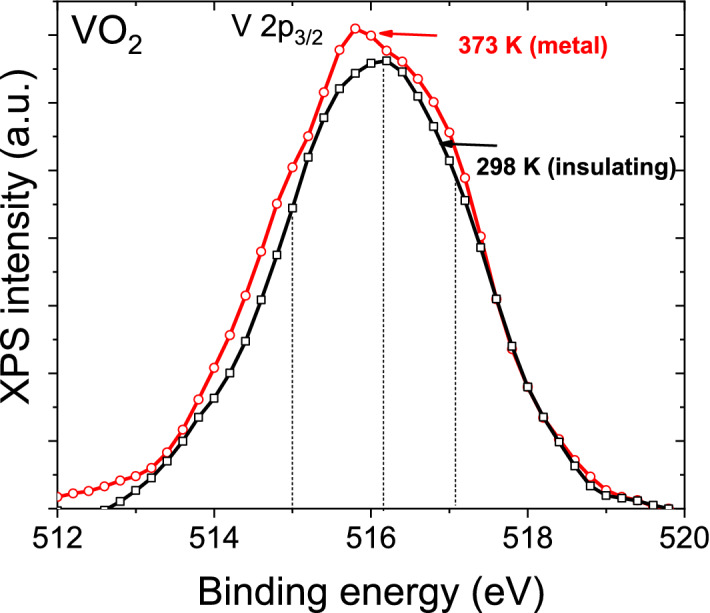
V 2p_3/2_ core-level XPS spectrum of stoichiometric VO_2_ taken above and below the transition temperature, after correction for the background.

In addition, a strong increase in the peak broadening can be seen in stoichiometric VO_2_ due to temperature-induced phase transition from insulating to the metallic phase at temperatures higher than T_IMT_ (Fig. 4). An increase in the signal at both low and high BE can be seen, indicating the formation of V^3+^ and V^5+^ cations. Localization of itinerant electrons at the V^4+^ site in the metallic state would lead to V^3+^ cation (polaron) formation. Therefore, this broadening seen is likely due to changes in the initial state. An explanation for the formation of V^5+^ is given in “Discussion” section. Supplementary Tables S1 and S2 summarizes the various fitting parameters along with the values reported in the literature for single crystalline and polycrystalline samples.

## Discussion

### A cooperative structural and electronic phase transition model

The combined results of electrical resistivity and photoemission measurements show that the doping-induced IMT process is controlled by the lattice V^3+^/V^5+^ ratio. An increase in V^3+^ concentration in the lattice relative to stoichiometric lattice decreases T_IMT_, while an increase in V^5+^ leads to an increase in T_IMT_. Further, if increased peak broadening seen in the XPS is due to initial state effects, then the results show that the temperature-induced IMT process in stoichiometric VO_2_ results in the formation of V^3+^ and V^5+^ cations in the metallic phase. While this is not conclusive evidence, we show below that other evidence exists in the literature that substantiates this assertion. Thus, any proposed model must account for both doping- and temperature-induced IMT and structural transitions.

Based on the XPS results, we propose that in stoichiometric VO_2_, strong charge fluctuation occurs in the metallic phase, which leads to the creation of e^−^ and h^+^ pairs that correspond to the formation of V^3+^ and V^5+^ cations between the V–V dimers seen in the insulating ground state. This implies that in the metallic phase, the LHB $d_{\parallel }$ band corresponds to a V^5+^ hole band (3d^0^ configuration) that lacks the 3d^1^ electron of the V^4+^ cations. The antibonding $d_{\parallel }$ band ($d_{\parallel }^{*}$) corresponds to a filled V^3+^ band of 3d^2^ configuration. In the metallic phase of stoichiometric VO_2_, charge fluctuation would lead to a V^3+^/V^5+^ ratio of 1. The conduction occurs through hopping of e^−^ in V^3+^ to the next site and so on through the lattice (polaronic hopping). Similarly, h^+^ in V^5+^ can also hop to the neighboring sites and move through the lattice, thus leading to hole conductivity. Since V^3+^ and V^5+^ cations can be considered as donors and acceptors, respectively, in a stoichiometric lattice, the metallic phase consists of both e^−^ and h^+^ and therefore, both contribute to the conductivity. This is supported by the results of transport studies and arguments presented by some earlier researchers. While both hall-effect and thermopower measurements indicate dominant conduction by electrons in both phases, the measured electron mobility in both phases is low (~ µ ~ 0.5 cm^2^ V s^−1^)^50^ and the measured Hall coefficient is nearly three-times lower than that expected for a simple metal with one electron per atom. Both theoretical^51^ and experimental results^52,53^ show very large electron effective mass with values of m* between 2 and 65 times the free carrier mass (m^0^). These results together with the observed low Hall constant and short mean free path of ~ 20% of V–V distance led to the suggestion by Ladd^54,55^ of a two-band conduction model where both the e^−^ and h^+^ contribute to conductivity but their nearly equal but opposite polarity contributes to the lower calculated values of Hall constant and mobility. He suggested that true mobility may be higher than the calculated values. Kabashima et al*.*^56^ indicated that both band and hopping conductivity is present in the metallic phase, and therefore models with a single type of charge carrier is inappropriate to explain the changes. Similarly, theoretical calculations of Caruthers and Kleinman^51^ indicate that stoichiometric VO_2_ to be intrinsic with the presence of both e^−^ and h^+^ in the lattice. Zylbersztejn and Mott indicated based on the results of thermoelectric power measurements of VO_2_ that it is characteristic of intrinsic regime^3^. It is also likely that holes in the narrow LHB may form immobile or low mobility small polarons, and therefore, its contribution to conductivity is lower than that of electrons.

**Figure 5:**
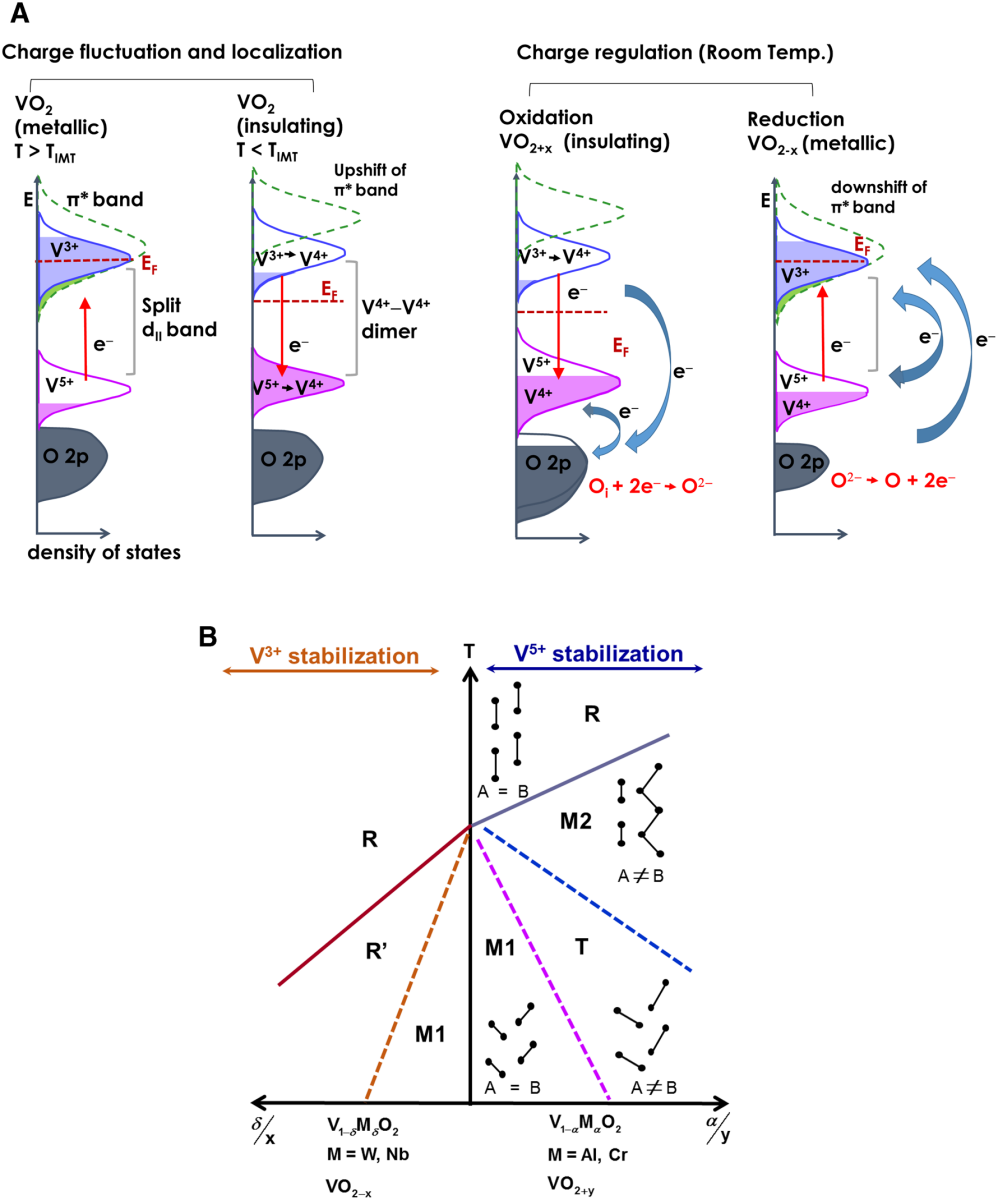
(**A**) Schematic showing the electronic aspects of the charge fluctualtion and charge localization processes in the metallic and insulating phases of VO_2_, respectively. Also shown is the charge regulation occuring in doped VO_2_ as a result of charge injection and its effect on IMT process; and (**B**) phase diagram of structural evolution of VO_2_ as function of doping and oxygen stoichiometry of the lattice. Doping with acceptor-type impurity or defect lead to the stabilization of V^5+^ defect states, which then stablizes the various insulating monoclinic phases. In contrast, doping with donor-type defects induce metallic phase transitions due to stablization of V^3+^ defect states.

At T < T_IMT_, the charge fluctuations seen in the metallic phase leading to V^3+^ and V^5+^ polaron formation is suppressed in the insulating phase. The low thermal energy coupled with the stronger correlation effects likely leads to the localization of a majority of e^−^ from V^3+^ sites at hole fields (V^5+^ sites), which would convert the sites from $V^{3 + } \to V^{4 + }$ and $V^{5 + } \to V^{4 + }$. Thus, we attribute the observed formation of V–V dimers in the insulating state to the charge localization between the fluctuating states to create V^4+^–V^4+^ dimers. Therefore, one can view the insulating ground state of VO_2_ as a self-compensated semiconductor, where most of donors are neutralized by the acceptors to create a low-conducting state consisting of a predominantly V^4+^ lattice sites and a small number of uncompensated V^3+^ and V^5+^ cations. This is shown schematically in Fig. 5A. Upon thermal excitation at the transition temperature, e^−^ transition from the lower lying filled acceptor states ($V^{4 + } \to V^{5 + }$) to the empty $d_{\parallel }^{*}$ and $\pi^{*}$ donor states ($V^{4 + } \to V^{3 + }$) should result in an increased filling of higher energy states (lower BE states in VB spectrum), an increase in the DOS of both V^3+^ and V^5+^ states, and a corresponding decrease in the DOS of V^4+^. This would explain the spectral weight transfer of the V 3d^1^ VB spectra towards E_F_. Further, fast e^−^ hopping between two polaronic V^3+^ and V^5+^ sites in the metallic phase should result in a more symmetric structure (equally spaced vanadium chains), while carrier trapping in the low-temperature phase leads to structural distortion. The above picture represents the possible electronic aspect of the IMT process. However, there are other important aspects of the transition that must be considered. For instance, in the metallic state, there would be a strong Coulomb interaction between the e^−^ and h^+^ pairs created by the fluctuation which would result in a bound state and not contribute to the conductivity. Combined XAS and XPS studies indicate a closing of 0.6 eV gap upon transition to the metallic phase^6^, which is much larger than the energy scale of the T_IMT_ (kT = 0.03 eV). Further, in a simple donor–acceptor band model, one would expect a continuous e^−^ excitation with increasing temperature instead of the sharp transition at a critical temperature with a large latent heat of phase change (1020 cal/mole^57^). The contribution of electronic entropy of large density of states of the metallic phase alone cannot account for the measured latent heat, as pointed out by Ladd^54^. These discrepancies can be resolved if one considers a co-operative structural and electronic transition process. In our model, the metallic state with co-existing e^−^ and h^+^ charges are stabilized by screening of the $d_{\parallel }^{*}$ electrons against the Coulomb field of the $d_{\parallel }$ hole band by electron in a comparatively wider $\pi^{*}$ band, a concept proposed previously by Mott et al.^3,58^. In our model, the nucleation of an insulating state is a result of displacement of V atoms perpendicular to c-axis that specifically raises the energy of the $\pi^{*}$ band, thereby increasing the energetic gap between the $\pi^{*}$ band and $d_{\parallel }^{*}$ band. This removes the necessary screening between the V^3+^ $d_{\parallel }^{*}$ electrons and V^5+^ $d_{\parallel }$ hole band and causes localization of e^−^ in the Coulomb field of holes. This assertion is compatible with the results of Tanaka^59^, whose calculations performed using three-band Hubbard model showed that only necessary parameter for inducing phase transition is energy separation (D) between the $d_{\parallel }^{*}$ and $\pi^{*}$. Therefore, we believe that IMT is a cooperative process between temperature-induced lattice distortion, the resulting modulation of the energy of the $\pi^{*}$ band and its influence on the efficacy of screening of $d_{\parallel }^{*}$ electrons against the hole field.

In our two-band picture shown in the Fig. 5A, there is no collapse of the gap between the $d_{\parallel }^{{}}$ and $d_{\parallel }^{*}$ band, rather metallicity is induced by the downward shift of the $\pi^{*}$ band and consequent overlap with the $d_{\parallel }^{*}$ band. Optical measurements^52^ on high-quality VO_2_ show a low energy peak at ~ 1.1 eV due to $d_{\parallel }$ to $d_{\parallel }^{*}$ transitions, and a peak around 3.1 eV, due to O 2p-d band electronic transitions. Our temperature-dependent measurement of optical spectrum of VO_2_, as shown in Supplementary Fig. S3, show is no significant difference in the spectral shape of the transmission curve between the metallic and insulating phases in the 300–1700 nm spectral range, and only changes in the transmittance values are observed. Both the p–d and d–d peaks occur at the same energy in both the insulating and metallic phases, which indicates there is no shift in the O 2p and d bands between the two phases. In addition, we note that if our assertion of the presence of V^5+^ hole band in the metallic state is correct, then the interpretation of the X-ray absorption spectroscopy (XAS) data needs to be reevaluated with theoretical calculations. For instance, the rising portion of the O K edge spectral curve is taken to be due to transitions to the unoccupied $\pi^{*}$ band^6,60–62^. However, this transition could also be due to electron transitions from core-level to the unoccupied states of V^5+^ hole band. This is supported by the observation that the intensity of this peak is higher in the metallic state than in the insulating state, as reported in the literature^6,60,62^, and points to the lower occupancy of this band in the metallic state, which is opposite to conventional understanding of partly-filled $\pi^{*}$ band in the metallic state. Further, oxygen-stoichiometry-dependent XAS measurements of Yeo et al*.*^60^ show an increased spectral weight of this band with increasing O stoichiometry, which is consistent with our picture of an larger unoccupied V^5+^ hole band in the metallic and insulating phases of O-excess samples compared to that of O-deficient VO_2_.

The high-temperature phase of the VO_2_ is considered to be a bad metal because of its high relative resistivity. According to the Drude model of metallic conduction, electrical conductivity should decrease with an increase in the temperature because of the increased carrier scattering from the thermally-excited lattice vibrations. Transport measurements^53,63^ of the metallic phase, however, show that carrier mobility increases, albeit slowly, as the temperature is increased from T_IMT_. As thermal excitation facilitates polaronic hopping between neighboring sites, the trend in the carrier mobility supports the polaronic model of the metallic phase.

By accurately fitting the observed asymmetric ^51^V NMR spectral line of insulating VO_2_ with superposition of symmetrical lines of V^3+^ and V^5+^, Boyarsky et al.^19^ suggested that the low temperature phase of VO_2_ consists of V^3+^ and V^5+^. In our model, it is the high-temperature phase that consists of co-existing V^3+^ and V^5+^ cations. Our model, in some sense, is consistent with the donor–acceptor (D-A) pair model proposed by Berglund and Guggenheim^57^. While asserting that correlation effects are not important, the authors attributed the insulating phase at room temperature to a heavily compensated n-type semiconductor due to the presence of a high density of both “donor-like” and “acceptor-like” unidentified states within the band gap of VO_2_. Their argument for the existence of co-existing donor and acceptor states within the band gap stems from the observations that: (1) no clear abrupt optical absorption threshold has been observed in VO_2_ at the photon energy corresponding to the energy gap in the semiconducting state; (2) absence of any observable photoconductivity in VO_2_, and (3) absence of rectifying behavior at the metal–semiconductor (VO_2_) contact with high work function metals such as Pt, Au, Al or Cu. They argued that all of the above could be explained by the presence of a high density of trap (acceptor) states within the band gap. The model proposed in this study, though it differs from that of Berglund and Guggenheim^57^ in several aspects, fits with their concept of co-existing e^−^ and h^+^ states and the experimental observations stated above underlying their model.

Finally, we show that both doping-induced and stress-induced IMTs can be rationalized easily within the proposed mixed-valent (D-A) lattice framework. The schematic of the atomic arrangement, as shown by Pouget et al*.*^29^ of the two interpenetrating sublattices of vanadium atom chains, A and B, in different VO_2_ phases is shown Fig. 5B for illustrating the process of structural transitions. The oxygen octahedron, whose principle axis points in the [110] direction, surrounding each V atom of the sublattices are not shown for clarity. Starting from the stoichiometric monoclinic M1 phase of VO_2_, which is characterized by alternating shorter and longer V–V bond lengths and twisted V–V chains, we first show the effects of hole doping. Introduction of $O_{i}$ defects or substitutional trivalent dopants such as Cr, Al, and Fe into the M1 lattice will lead to the formation of O 2p holes or V^5+^ cations to maintain charge neutrality^29,64^. Electronically, the width of V^5+^ hole band will increase causing greater localization of any uncompensated e^−^ of the M1 phase, resulting in increased resistance of the lattice and a lower density of V^4+^ states in the $d_{\parallel }$ LHB due to its partial conversion to V^5+^ states. This is consistent with the lower DOS observed in the VB spectra with increasing O stoichiometry (Fig. 2). Structurally, the presence of V^5+^ would lead to a reduction in V–O bond length between the V^5+^ and the O^2−^ as well as the apical O^2—^O^2−^ distance in the surrounding oxygen octahedron. This contraction in the [110] direction would lead to an increase in the V–V distance in the [100]_R_, which causes a straightening of the tilted V–V pairs in the A sublattice, which in turn induces the depairing of V–V dimers in B sublattice to produce zig-zag chains, an arrangement defined as the M2 phase. Application of uniaxial stress in the [110]_R_ direction of M1 phase in undoped VO_2_ has the same effect of compressing the O–O bond distance as the hole-induced O–O shortening, thus both induce and stabilize insulating M2 and T phases^65^. The resultant increase in the V–V distance leads to a decrease in the band width of V^3+^ donor band and V^5+^ acceptor band and an increase in the energy separation between them, which would necessitate a greater thermal and phonon coupling for IMT process. Hence, an increase in T_IMT_ is observed. The results of Villenauve et al*.*^64^ who showed that stability of the M2 phase is closely linked to the oxygen stoichiometry of VMO_2+y_ lattice. The M2 phase disappears for $y \le - 0.01$, i.e*.* it is unstable in the presence of V^3+^ defects. In contrast, its stability domain increases when O stoichiometry is greater than 2. In other words, M2 phase is stabilized by V^5+^ defects, a result which is consistent with our proposed V^3+^–V^5+^ model.

Conversely, an increase in the O–O bond length and the resulting decrease in V–V bond distance would correspond to the case of increased band width (higher DOS) or lower energy gap between V^3+^$d_{\parallel }^{*}$ band and V^5+^$d_{\parallel }$ band, which should decrease T_IMT._ This is seen in epitaxial strained films^61^, where strong reduction in T_IMT_ has been shown with the application of stress in the [001] direction. This is also the case when $\ddot{V}_{O}$ defects (through hydrogenation and electrochemical gating^66^) or pentavalent or hexavalent substitutional impurities such as Nb, Mo, W, and Re are introduced in the lattice which leads to an increase in the V^3+^ states^28^. In all the above cases, the V^3+^ band stabilizes the rutile phase of VO_2_.

The above explanation for doping-induced IMT shows that T_IMT_ is regulated in the VO_2_ lattice through combined effects of charge redistribution between the V^3+^, V^4+^ and V^5+^ cations, and corresponding changes in the V–O and V–V bond lengths.

### Charge disproportionation in ternary vanadates: similarities and differences

Charge disproportionation has been noted in other correlated systems like ternary and quaternary alloys of vanadates involving substitution with lanthanides (LaVO_3_) or alkaline earth elements (La_1−x_Ca_x_VO_3_, Sr_1−x_Ca_x_VO_3_). In these systems substitution of trivalent La with divalent elements such as Ca or Sr leads to paramagnetic metallic hole doping of LaVO_3_ for x ≥ 0.2, which is otherwise an antiferromagnetic Mott insulator for 0 < x < 0.2. Photoemission studies^67–69^ with different excitation energies have shown that the measured electronic properties strongly depends on the escape depth with the surface region exhibiting charge localization or insulating behavior while the bulk of the film corresponding to the metallic phase, as noted by the absence/presence of coherent signal near E_F_. Maiti et al*.*^67,68^ attributed this to the enhanced correlation effect at the surface compared to bulk that causes spontaneous disproportionation of lattice V^4+^ ions according to the reaction $$2V$$$$^{4 + } \to V^{3 + } + V^{5 + }$$. Consistent with this reaction, all members of the series show three distinct peaks in the V 2p_3/2_ core-level spectra corresponding to V^3+^, V^4+^, and V^5+^ species. Further, the observation of 1:1 ratio of V^3+^ to V^5+^ peak intensities in these compounds together with an observed increase in their signal when probed with lower energy excitation (more surface sensitive) provided strong evidence for spontaneous electronic phase segregation into metallic bulk consisting of V^4+^ cations and a more insulating surface with V^3+^ and V^5+^ cations. Therefore, such vanadates serve as an important reference for understanding the effects of charge disproportionation reactions in pure VO_2_ discussed in the present study. While the two systems may appear to be similar, they, however, differ in several aspects. The major difference between the two systems is the condition under which charge disproportionation occurs. Surface charge disproportionation into V^3+^ and V^5+^ has been attributed to be the cause for the formation of insulating phase in doped vanadates. In contrast, we attribute charge disproportionation (or fluctuation) into V^3+^ and V^5+^ in the bulk of the metallic phase of stoichiometric VO_2_, while the localization of e^–^ at the V^5+^ sites in the bulk as the cause for the formation of insulating phase with V^4+^ cations. In vanadates, disproportionation gives rise to localized, non-interacting mixed valent states. In contrast, in stoichiometric VO_2_, the mixed valent states correspond to delocalized states and therefore contribute to conductivity. Further, our results across stoichiometrically-modulated VO_2_ indicate that the ratio of V^3+^/V^5+^ dictates T_IMT_, i.e. an increase in V^3+^/V^5+^ ratio decreases T_IMT_ and vice versa. The underlying mechanism giving rise to these differences in not clear. Although both VO_2_ and SrVO_3_ represent d^1^ configuration, according to Fujimori et al*.*^38^ the ratio of effective Hubbard parameter U_eff_ to band width (W) of VO_2_ is expected to smaller than that of SrVO_3_, as W is larger for VO_2_ due to much shorter V–V distances. Therefore, correlation effects are expected to stronger in SrVO_3_ than in VO_2_, thus leading to strong charge localization. Another interesting feature is that the V 2p_3/2_ peaks of doped vanadates show distinct peaks across the entire compositionally-modulated series that are clearly resolvable into Gaussian peaks corresponding to V^3+^, V^4+^, and V^5+^ signals. In contrast, spectrum of stoichiometric VO_2_ in both metallic and insulating states shows a single broad peak that can be easily fitted by a single Gaussian peak albeit with different FWHM between the two phases. This indicates that the nature of ground state, whether it is localized or delocalized valence states, between the two systems is different^48,49^. Secondly, charge disproportionation in vanadates is restricted to the surface, as seen from the differences in the measured density of states between He-I, He-II and Al K_α_ excitation signal. In all compositionally tuned VO_2_ samples, the measured VB structure using Al K_α_ line is consistent with the bulk transport properties and, therefore, are representative of bulk electrical phase transitions. Further studies are needed to gain a deeper understanding of the differences in the two systems.

In conclusion, the results of oxygen stoichiometry-dependent phase transitions presented here offers a new perspective on the mechanism of temperature-and doping-induced IMT process. Results and their interpretations suggest that charge fluctuation in the metallic phase of intrinsic VO_2_ results in the formation of e^−^ and h^+^ pairs in that lead to polaronic V^3+^ and V^5+^ cation states. The $d_{\parallel }$ lower Hubbard consists of the V^5+^ hole band while the upper $d_{\parallel }^{*}$ and $\pi^{*}$ bands form the V^3+^ bands. The metal-to-insulator transition is linked to the cooperative effects of changes in the V–O bond length, localization of V^3+^ electron at V^5+^ sites that results in the formation of V^4+^–V^4+^ dimers, and removal of $\pi^{*}$ screening electrons. Our results on the doping-induced process suggests these co-operative changes are linked to the lattice V^3+^/V^5+^ concentrations of VO_2_, wherein an increase in V^5+^ concentration relative to V^3+^ suppresses the metallic phase (increases T_IMT_), while an increase in V^3+^/V^5+^ ratio suppresses the insulating phase (decreases T_IMT_). Lastly, analysis indicates that cooperative effects of structural and electronic phase transitions are needed to fully explain the IMT process.

## Methods

### Synthesis of VO_2_

Bulk thin films of VO_2_ were synthesized by reactive vapor transport by annealing commercial VO_2_ powder (Sigma Aldrich) at 650 °C in a quartz boat. Stoichiometric VO_2_ was deposited directly on a Si substrate kept on top of the boat. Two-dimensional VO_2_ platelets were synthesized using a two-step process involving reductive phase transformation of V_2_O_5_ platelet films. Single crystalline V_2_O_5_ platelets were synthesized on fluorinated tin oxide (FTO)-coated glass slides by hot filament chemical vapor deposition, as reported previously^26,70^. Briefly, the technique consists of resistively heating a vanadium wire in the presence of oxygen at a pressure of 800 mTorr at a substrate temperature between 450 and 500 °C. As prepared films were then annealed in a H_2_/Ar (1:4 vol ratio) atmosphere at a temperature of 540 °C for 4–8 h to enable conversion to VO_2_. The VO_2_ formed by this procedure was observed to be oxygen deficient and metallic. These films are referred to as “VO_2−x_” in the text. Metallic VO_2−x_ was converted to semiconducting VO_2_ by further annealing in air at 220 °C for 24–72 h to convert to stoichiometric VO_2_ phase. X-ray diffraction pattern of both phases were indexed to a pure VO_2_ phase.

### Inductively coupled plasma mass spectroscopy (ICP-MS) analysis

The stoichiometry of vanadium oxide samples was determined using combined thermogravimetric analysis (TGA) and ICP-MS analysis. A known amount of oxide sample was weighted in the TGA balance. The sample was then heated up to a temperature of 220 °C under nitrogen and the mass loss due to physisorbed water was recorded, which was generally 3–5% of the sample weight. The powder was then dissolved in concentrated HCl solution and then diluted by a factor of 1:20. The vanadium concentration in this solution was then determined using a Bruker 820 ICP-MS spectrometer by comparison with standard solutions having vanadium concentrations of 0.3 ppm, 1 ppm and 3 ppm. The oxygen content of the powder was determined from the relative mass difference between the total weight corrected for ambient adsorbates from the TGA analysis and the weight of vanadium determined from ICP-MS analysis.

### Electrochemical doping

Electrochemical doping of the oxide was performed using a three electrode setup with platinum as the counter electrode and silver foil as the reference electrode in 0.1 M solution of tetrabutylammonium triple salt of hydrogen monopersulfate ($2HSO_{5}^{ - }$), peroxomonosulfate ($HSO_{4}^{ - }$), and persulfate ($SO_{4}^{2 - }$) (tradename OXONE) in propylene carbonate (PC) under ambient conditions. For all measurements, charging was performed by applying a potential step using a potentiostat (660E CH Instruments) for a fixed time period or until ionic equilibrium throughout the bulk of the film was reached, which was indicated when the charging current reached low values (~ 10^−6^ to 10^−7^ A). Electrical resistance of the VO_2_ film was then measured using two molybdenum clips pressed onto the sample and a Keithley 2400 Sourcemeter. The nature of dominant carriers in some of the samples was determined using Hall Effect measurements (Silicon Valley Science Labs) at a magnetic field of 8000 Gauss.

### Calibration of reference scale

The potential of the Ag pseudo-reference electrode was calibrated using ferrocene/ferrocenium redox couple under working conditions using a polished Pt disk as the working electrode, Pt wire as the counter electrode and Ag foil as the reference electrode. All potentials are referenced with respect to the standard hydrogen electrode (SHE) that has a work function of ~  − 4.44 eV with respect to the vacuum level.

### Optical studies

Raman spectrum of VO_2_ films was recorded using a HORIBA Scientific LabRAM HR Evolution spectrometer at an excitation wavelength of 633 nm (He–Ne laser). Raman spectra of electrochemically-doped electrodes were collected ex-situ from the emmersed electrodes after drying under vacuum.

### Photoemission studies

XPS measurements were done using a Physical Electronics PHI 5000 Versa Probe system using a monochromatic Al (Κ_α_) (1486.7 eV) X-ray source and detected using a 150 mm radius hemispherical electron energy analyzer. All samples were gently sputtered with an Ar^+^ beam for 2 min to remove any over-oxidized surface layer, and then annealed in ultrahigh vacuum at 220 °C for 3 h to desorb physisorbed electrolyte or other contaminants. Calibration of the spectrometer was performed using silver and gold standards. During XPS measurements with VO_2_, the Fermi edge of the spectrum was calibrated against the signal from a polycrystalline Ag foils that was cleaned in vacuum using Ar^+^ sputtering. Survey scans were collected over the range from 1100 to 0 eV with a pass energy of 117.4 eV for collection duration of ~ 10 min. Higher-resolution scans were collected over a range of 10 eV around the peak of interest for ~ 30 min.

## Supplementary information

Supplementary Information 1

## Data Availability

The data that support the findings of this study are available from the corresponding author upon reasonable request.
