# Supplementary material for: Effects of charge fluctuation and charge regulation on the phase transitions in stoichiometric VO2
Source: Sci Rep. 2020 Oct 13;10:17121. doi: 10.1038/s41598-020-73447-9 (PMC7553960; doi:10.1038/s41598-020-73447-9)
Supplement: Supplementary file 1 — Supplementary Information 1 [file 41598_2020_73447_MOESM1_ESM.pdf]

Supporting Information

Effects of Charge Fluctuation and Charge Regulation on the Phase Transitions in Stoichiometric VO_2

Siddharth Joshi,[a*] Nicholas Smieszek,[a*] and Vidhya Chakrapani,[a,$,#]

[a] Howard P. Isermann Department of Chemical and Biological Engineering

[$] Department of Physics, Applied Physics, and Astronomy

Rensselaer Polytechnic Institute, Troy, NY-12180

*Equal contribution

[#] Corresponding author: chakrv@rpi.edu

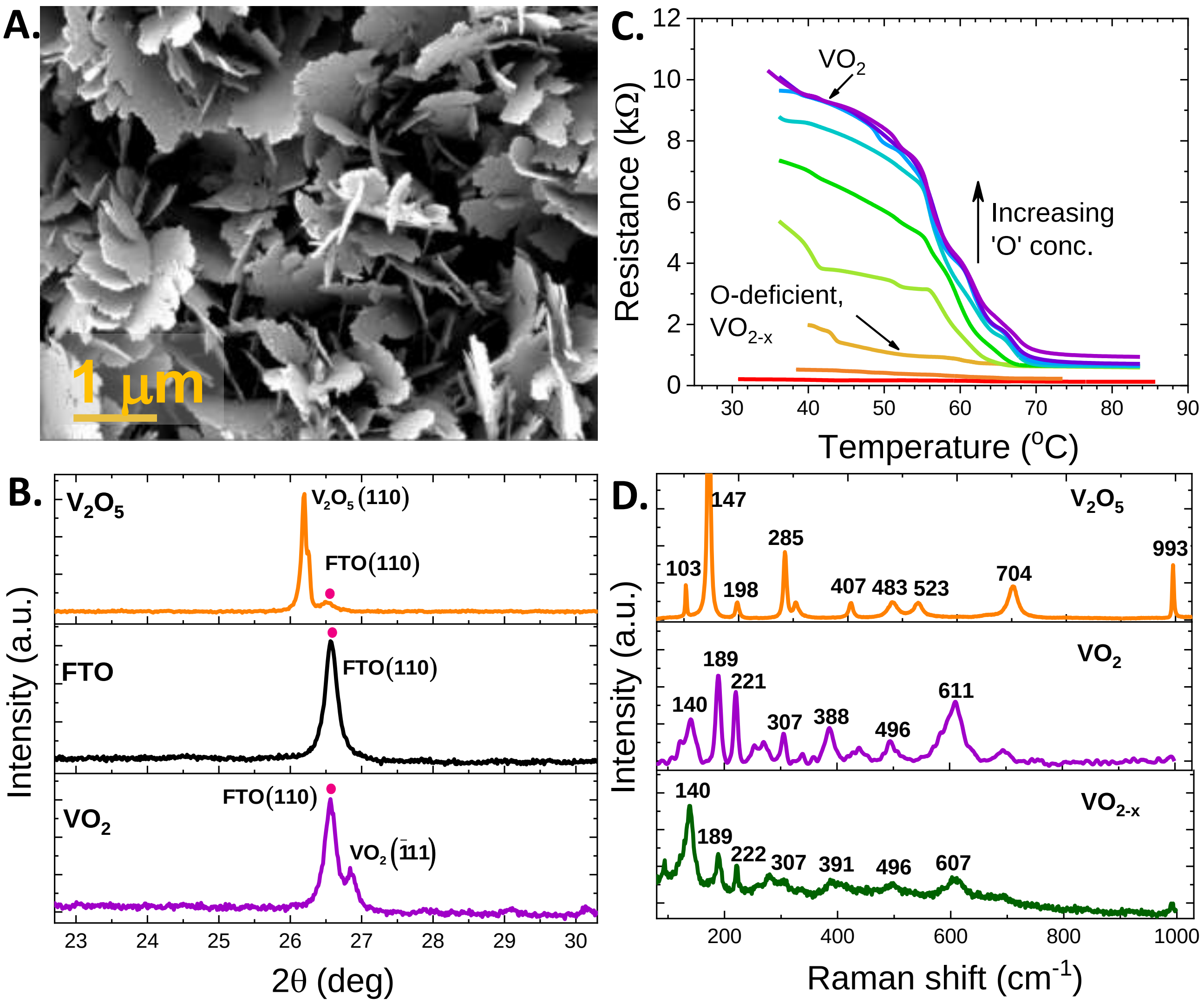

Figure S1A) An SEM image of the synthesized VO_2 platelets prepared using hot-filament chemical vapor deposition; B) XRD patterns of V_2O_5, FTO-coated glass substrate, and monoclinic VO_2; C) Changes in the resistance of VO_2 of various stoichiometry during heating between room temperature and 100 °C; and D) Raman spectrum of oxygen deficient (VO_{2-x}) and stoichiometric VO_2 confirming the structure as monoclinic M1 phase.

Table S1. Comparison of XPS fit parameters for $V2p_{3/2}$ and O 1s peaks of VO_2 and V_2O_5 measured in this work and those reported in the literature. Fwhm = full-width-at-half-maximum.

**Nature of Oxide**	**BE (eV)** $V2p_{3/2}$	**fwhm (eV)** $V2p_{3/2}$	**BE (eV)** $O1s$	**fwhm (eV)** $O1s$	Δ **(eV)** $(BE_{O1s} - BE_{V2p_{3/2}})$	**Ref.**
**VO_2**						
**Single crystal platelets**	**516.1**	**2.8**	**530**	**1.5**	**13.9**	**This work**
Single crystal @ RT (UHV cleaved)	516.2	3.2	529.9	1.8	13.7	Sawatzky & Post[1]
@ 373 K	515.9	4.1	529.7	2.0		
Commercial powder (sputtered+ annealed)	515.95	3.3	529.4	1.2	13.45	Hryha *et al.*[2]
Single crystal (UHV cleaved)	516	1.95	-	-	-	Demeter *et al.*[3]
Polycrystalline	515.65	4.0	530	2.8	14.35	Mendialdua *et al.*[4]
Thin film	515.5	3.0	-	-	-	Cui *et al.* [5]
**V_2O_5**						
**Single crystal platelets**	**517.2**	**1.1**	**530.0**	**1.5**	**12.8**	**This work**
Single crystal (UHV cleaved)	517.2	1.2	-	-	-	Demeter *et al.*[3]
Polycrystalline	517.0	1.3	529.8	1.75	12.8	Mendialdua et al.[4]
Single crystal crushed	516.9	1.6	529.8	1.7	12.9	Sawatzky & Post[1]
Single crystal	517.2	1.2	-	-	-	Demeter et al.[3]

Table S2. Summary of XPS fitting analysis of V $2p_{3/2}$ and O 1s multiplet peaks of stoichiometric and non-stoichiometric vanadium dioxide.

V_2O_5

Peak ID	***V $2p_{3/2}$ BE (eV)***	***V $2p_{3/2}$ fwhm (eV)***	***%Area V peaks***	***Peak ID***	***O 1s BE (eV)***	***O 1s fwhm (eV)***	***%Area O peaks***
V^{4+}	515.8	1.3	10.3	O^{2-}	530.0	1.3	84.2
V^{5+}	517.2	1.1	89.7	-OH	531.7	2.4	15.8
Effective oxidation state: **+4.9**							
**V_2O_{5-x}**							
V^{4+}	515.7	1.1	15.86				
V^{5+}	517.2	1.3	84.13				
Effective oxidation state: **+4.84**							

$VO_{1.86}$

Peak ID	***V $2p_{3/2}$ BE (eV)***	***V $2p_{3/2}$ fwhm (eV)***	***%Area V peaks***	***Peak ID***	***O 1s BE (eV)***	***O 1s fwhm (eV)***	***%Area O peaks***
V^{3+}	515.03	2.24	44.27	O^{2-}	530.0	1.5	81.0
V^{4+}	516.1	1.8	35.93	-OH	531.5	1.5	15.0
V^{5+}	517.2	1.9	19.85	$-H_2O$	532.9	2	4.0

Effective oxidation state: **+3.76**

VO_2

Peak ID	***V $2p_{3/2}$ BE (eV)***	***V $2p_{3/2}$ fwhm (eV)***	***%Area V peaks***	***Peak ID***	***O 1s BE (eV)***	***O 1sfwhm (eV)***	***%Area O peaks***
V^{3+}	515.03	2.1	33.0	O^{2-}	530.0	1.4	87.9
V^{4+}	516.1	1.8	38.0	-OH	531.4	1.0	10.0
V^{5+}	517.2	1.9	29.0	$-H_2O$	532.6	2	2.1

Effective oxidation state: **+3.96**

$VO_{2.06}$ (Electrochemical-OXONE)

Peak ID	***V $2p_{3/2}$ BE (eV)***	***fwhm (eV) V $2p_{3/2}$***	***%Area V peaks***
V^{3+}	515.03	2.1	25.8
V^{4+}	516.1	1.8	35.8
V^{5+}	517.2	2.23	38.4

Effective oxidation state: **+4.13**

Table S3. Summary of stoichiometry of various vanadium oxide samples used in the present study determined from the ICP-MS and XPS analysis.

**Samples**		**O/V ratio**		**Average V Oxidation State**	
		**ICPMS**	**XPS (from V valence peaks)**	**ICPMS**	**XPS**
**Sample -1**	V_2O_5	2.44	2.45	+4.88	+4.9
**Sample -2**	VO_{2+y} -1	-	2.063	-	+4.13
**Sample-3**	VO_{2+y} -2	-	2.21	-	+4.43
**Sample-4**	VO_2	1.967	1.98	+3.93	+3.96
**Sample-5**	VO_{2-x}	1.86	1.88	+3.72	+3.76
**Sample-6**	VO_{2-x}	1.76	-	+3.52	-

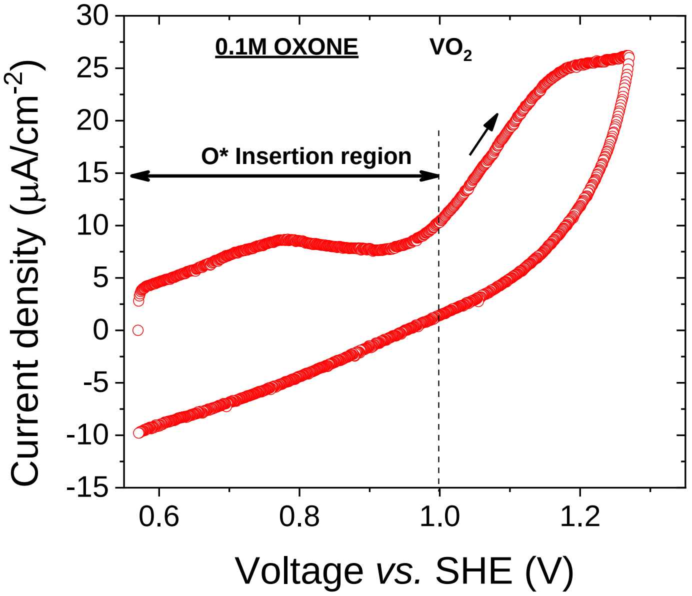

Figure S2. Cyclic voltammogram of VO_2 electrode during oxidative potential cycling in air-saturated OXONE electrolyte. The potential region of O insertion is indicated.

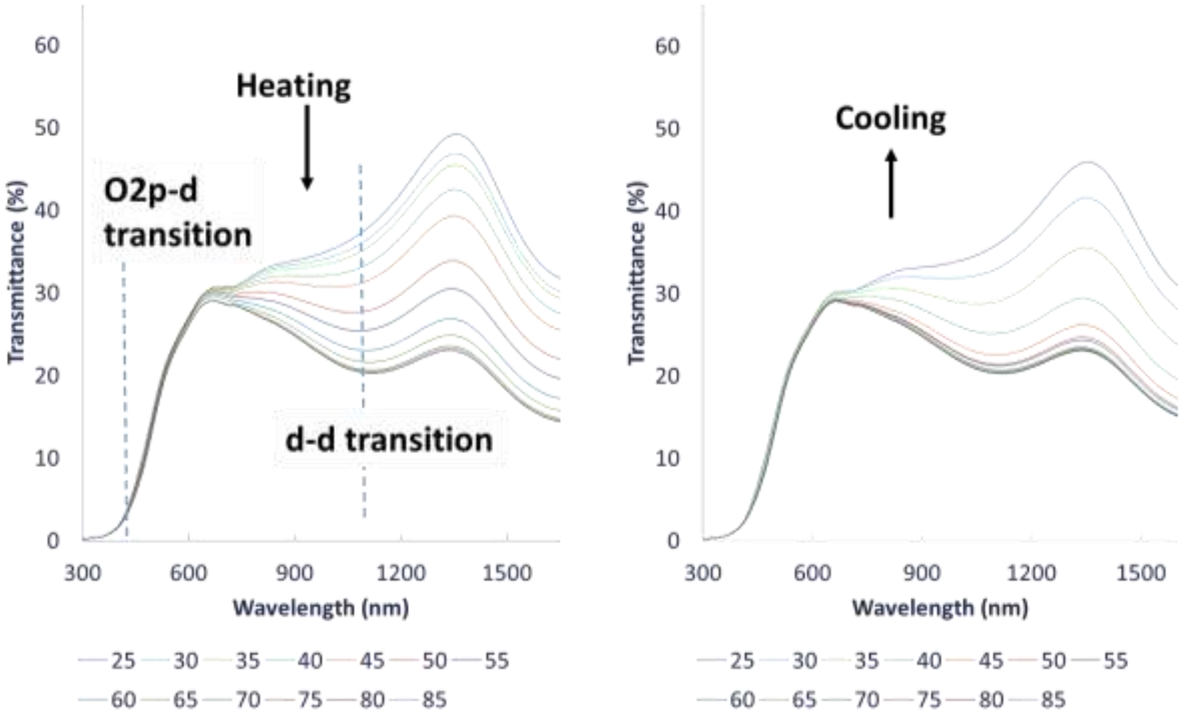

Figure S3. Optical transmittance spectra of stoichiometric VO_2 taken at various temperatures from 25 ºC to 85 ºC during heating and cooling.

REFERENCES

1 Sawatzky, G. & Post, D. X-ray photoelectron and Auger spectroscopy study of some vanadium oxides. *Phys. Rev. B* **20**, 1546, (1979).

2 Hryha, E., Rutqvist, E. & Nyborg, L. Stoichiometric Vanadium Oxides Studied by XPS. *Surf. Interface Anal.* **44**, 1022-1025, (2012).

3 Demeter, M., Neumann, M. & Reichelt, W. Mixed-valence vanadium oxides studied by XPS. *Surf. Sci.* **454**, 41-44, (2000).

4 Mendialdua, J., Casanova, R. & Barbaux, Y. XPS studies of V_2O_5, V_6O_{13}, VO_2 and V_2O_3. *J. Electron. Spectrosc. Relat. Phenom.* **71**, 249-261, (1995).

5 Cui, J., Da, D. & Jiang, W. Structure characterization of vanadium oxide thin films prepared by magnetron sputtering methods. *Appl. Surf. Sci.* **133**, 225-229, (1998).
